# A Study on Systematic Improvement of Transformer Models for Object Pose Estimation

**DOI:** 10.3390/s25041227

**Published:** 2025-02-18

**Authors:** Jungwoo Lee, Jinho Suh

**Affiliations:** 1 Smart Mobility Research Center, Korea Institute of Robotics and Technology Convergence (KIRO), Pohang 37666, Republic of Korea; ricow@kiro.re.kr; 2 Major of Mechanical System Engineering, Pukyong National University, Busan 48513, Republic of Korea

**Keywords:** transformer, object pose estimation, low-rank weight decomposition

## Abstract

Transformer architecture, initially developed for natural language processing and time series analysis, has been successfully adapted to various generative models in several domains. Object pose estimation, which uses images to determine the 3D position and orientation of an object, is essential for tasks such as robotic manipulation. This study introduces a transformer-based deep learning model for object pose estimation in computer vision, which determines the 3D position and orientation of objects from images. A baseline model derived from an encoder-only transformer faces challenges with high GPU memory usage when handling multiple objects. To improve training efficiency and support multi-object inference, it reduces memory consumption by adjusting the transformer’s attention layer and incorporates low-rank weight decomposition to decrease parameters. Additionally, GQA and RMS normalization enhance multi-object pose estimation performance, resulting in reduced memory usage and improved training accuracy. The improved model implementation with an extended matrix dimension reduced the GPU memory usage to only 2.5% of the baseline model, although it increased the number of model weight parameters. To mitigate this, the number of weight parameters was reduced by 28% using low-rank weight decomposition in the linear layer of attention. In addition, a 17% improvement in rotation training accuracy over the baseline model was achieved by applying GQA and RMS normalization.

## 1. Introduction

Transformer architecture [1] was designed to solve natural language processing and time series sequencing problems. It has also been applied to generative models in various domains, such as computer vision captioning [2,3,4], object classification [5,6], and object detection [7,8,9]. This architecture has shown excellent performance as a complement to existing RNN and CNN-based models.

Object pose estimation is the accurate determination of the three-dimensional position and orientation of an object. This technique provides a computational understanding of the pose of a given object using image or sensor data, often from cameras. Accurate objects pose information is critical for the perception and interaction of robotic systems with their environment, enabling tasks such as object manipulation. In addition, object pose estimation is fundamental for safe navigation by facilitating recognition of surrounding vehicles, pedestrians, and traffic lights in the context of autonomous driving.

Traditional methods for object pose estimation, which rely on feature point extraction and model matching algorithms, often struggle with robustness in a variety of environments, especially those characterized by varying illumination and occlusion. Recently, deep learning-based models [10,11,12,13] have been used to predict the pose of objects directly from images.

In this study, we present a transformer-based model for object pose estimation and demonstrate that systematically optimizing each component of the model architecture not only minimizes hardware resource requirements, such as GPU memory, but also improves estimation accuracy through efficient design.

The main contributions of this study are summarized as follows:

- We introduce a baseline model that uses a transformer for object pose estimation. In the transformer architecture of this model, input data consisting of a 3D point cloud derived from object information is serialized. In addition, position encoding for embedding the input data is omitted since it is not affected by considerations in the temporal domain.
- To improve the performance of the transformer model for object pose estimation, the following modifications are introduced: (i) the output dimension in the attention layer of the transformer is expanded to support multiple object estimation; (ii) the input weight matrices in the attention layer are approximated by two smaller matrices, reducing the total number of model parameters; and (iii) multi-head attention is replaced by grouped query attention (GQA) [14,15], RMS normalization is incorporated instead of traditional layer normalization, and the activation function is switched from ReLU to SiLU.

## 2. Related Works

In recent years, transformer architecture has attracted considerable attention in the field of natural language processing (NLP) due to its breakthrough performance. Transformers have achieved state-of-the-art (SOTA) results in various NLP tasks by overcoming the limitations of traditional recurrent neural networks (RNNs) in learning long-term dependencies and have been widely adopted. The core mechanism of transformers, self-attention, computes interactions between all words in an input sequence and assigns importance weights to effectively capture contextual information. Multi-head attention [16,17] extends this capability by applying multiple self-attention mechanisms in parallel. This allows the model to analyze input sequences from different representational subspaces. However, the transformer architecture lacks an inherent mechanism for explicitly modeling word order due to its reliance on parallel processing. To overcome this limitation, positional encoding is incorporated to provide the model with information about word positions. This allows the model to efficiently capture order-dependent semantic variation.

In this study, a transformer model with fundamental architecture was utilized. The transformer is a sequence-to-sequence model comprising an encoder and a decoder that processes an input sequence to generate an output sequence. The encoder consists of N identical layers, transforming the input sequence into a fixed-length vector representation. Each layer uses a multi-head self-attention mechanism and a feedforward network (FFN) to capture relationships between all words in the input sequence and perform nonlinear transformations. The decoder generates the output sequence using the context vectors generated by the encoder. It incorporates a masked multi-head self-attention mechanism and cross-attention to learn dependencies between previously generated words and interactions with the encoder output.

**Figure 1:**
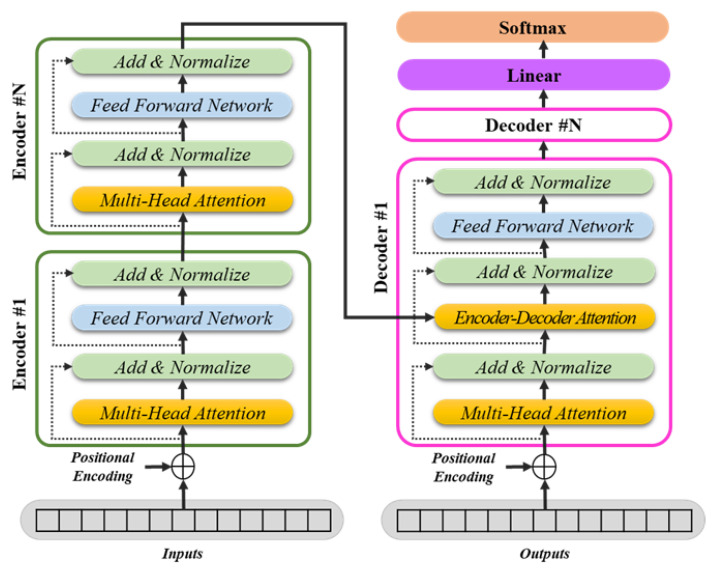
Architecture of the vanilla transformer.

The overall baseline structure of the transformer is shown in Figure 1 below.

Object pose estimation is a fundamental research area in computer vision. It focuses on determining the position and orientation of an object in three-dimensional space. This problem is typically formulated as the estimation of the six degrees of freedom, which have three translational and three rotational components. Accurate pose estimation is critical for a variety of applications, such as robotic manipulation, augmented reality, and 3D reconstruction. Recent research efforts have focused on improving the accuracy and robustness of pose estimation algorithms, especially in complex and challenging scenarios, by using deep learning and probabilistic frameworks.

In transformer-based pose estimation methods, the TransPose framework [18,19] employs a geometry-aware transformer encoder to extract local features from point clouds while simultaneously incorporating global context, thereby improving robustness to occlusions. CatFormer [20] improves category-level pose estimation through a multi-stage deformation process, which uses transformers to model geometric relationships and iteratively refine feature representations. For depth-based pose estimation, the SwinDePose approach [21] exclusively utilizes depth images, computing angles between normal vectors and coordinate axes. These features are then processed using a swin transformer to achieve accurate pose estimation. In addition, a novel end-to-end method [22] uses implicit representations to bridge discrete and continuous feature maps, thereby improving the accuracy of 6D pose estimation. This approach utilizes a compact dual-stream network to estimate object surface features, while singular value decomposition (SVD) is applied for rotation representation to further refine pose predictions. This technique has demonstrated promising performance on the LINEMOD benchmark.

## 3. Methods

### 3.1. Baseline Transformer Model for Single Object Pose Estimation

The proposed baseline transformer architecture for object pose estimation utilizes an encoder-only transformer to convert point cloud data into a feature vector, which is then processed by a linear multi-layer perceptron (MLP) to predict the object’s rotation and translation. Unlike sequential data, point cloud inputs represent spatial information without a temporal order, eliminating the need for positional encoding. Consequently, no positional encoding is required during the embedding process.

The vision foundation model (VFM) is used to perform object detection and mask segmentation on the color images of objects captured by the camera. For object detection, models such as DINO [23] and Yolo-World [24] are used, while the mask segmentation is performed using the Segment Anything Model (SAM) [25,26] and the Segment-Everything-Everywhere-All-At-Once (SEEM) [27].

The depth image captured by the camera contains a distance value for each pixel. The depth information is efficiently transformed into point cloud data using the pseudocode of the conversion algorithm, as shown in Table 1 below.

The ‘w’ and ‘h’ are each pixel number of width and height axis. The ‘pt_base’ array represents the homogeneous coordinates of each pixel in the image. The ‘ppxy’ array represents the principal point coordinates of the camera. The ‘fxy’ matrix represents the inverse of the camera’s intrinsic matrix, specifically the inverse of the focal lengths. The ‘pt_vertex’ array stores the 3D coordinates of each pixel in the camera coordinate system.

The conversion of depth images into point clouds can be affected by several sources of error. These include sensor noise, resolution limitations, occlusions, material surface reflectivity, and others. However, by using methods such as sensor calibration, noise filtering, adaptive thresholding, lens distortion correction, and post-processing techniques, these errors can be effectively reduced, resulting in more accurate and reliable point clouds.

**Figure 2:**
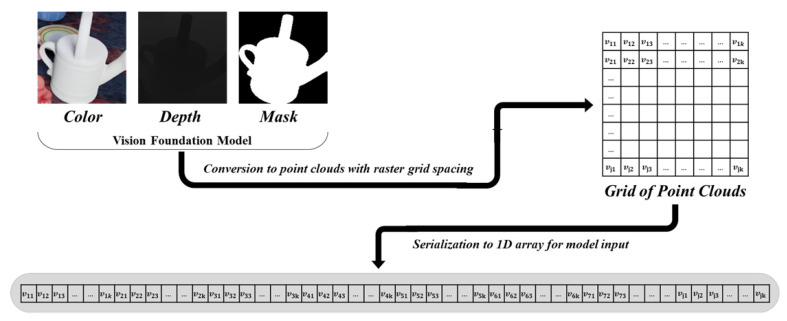
Process for extracting object information from camera images using VFM and generating one-dimensional model input data.

Using the location and mask information of the object detected by VFM, point clouds are generated that contain only the object with the background removed, along with the corresponding color data. The depth image of the object is segmented into a predefined number of grids in 2D space. For each grid cell, a representative depth value and an average color value are computed by averaging a subset of the included point clouds. These values are then organized into a two-dimensional matrix. Finally, each row of the matrix is concatenated to transform it into a one-dimensional sequence that serves as input to the transformer model. This process is illustrated in Figure 2.

**Figure 3:**
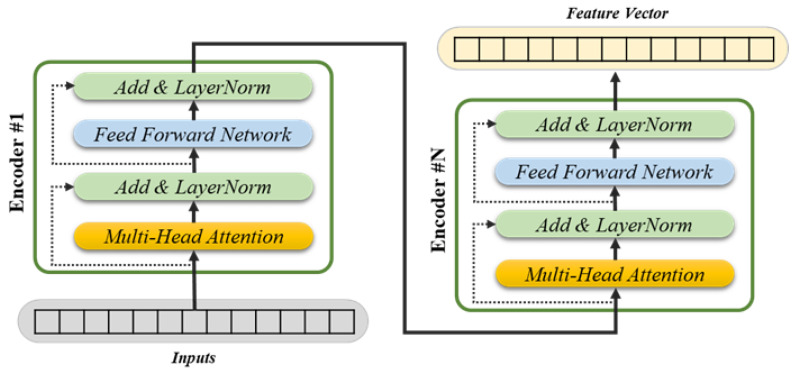
The baseline structure of the encoder-only transformer model.

A baseline transformer model for single object pose-estimation processes a one-dimensional serialized representation of the normalized point cloud data as an input and produces feature vectors as an output. This model is designed as an encoder-only transformer, adapted from the standard vanilla transformer architecture. During the encoding process, the residual connections incorporate the layer normalization, and the feed-forward network uses the ReLU activation function. The structure of this encoder-only transformer-based model is shown in Figure 3 below.

### 3.2. Extending the Matrix Dimension of the Attention Layer to Estimate Multi-Object Pose in a Single Prediction

The baseline model above uses a one-dimensional linearized input format for object pose estimation. To estimate the poses of multiple objects, a concatenated one-dimensional input vector must be fed into the model, and its length increases significantly. However, as the input vector grows, so does the memory required for matrix multiplication in the multi-head attention layer of the transformer. This leads to challenges in maintaining an appropriate batch size during training due to hardware constraints, and significantly increases the computational time required for processing.

To improve computational efficiency, we propose a novel approach to reduce memory consumption within the multi-head attention layer. We optimize matrix multiplication operations by restructuring the one-dimensional input vector into a two-dimensional matrix where the number of columns is equal to the number of objects.

The 3D tensor output of the traditional attention mechanism is reshaped into a 4D tensor by reducing the size of the last two dimensions of the output matrix. In the scaled dot product operation, matrix multiplication is performed on the last two dimensions of the multi-dimensional matrix, while the earlier dimensions are handled as batches. Thus, this approach effectively reduces both the memory space and computational load required for matrix multiplication.

**Figure 4:**
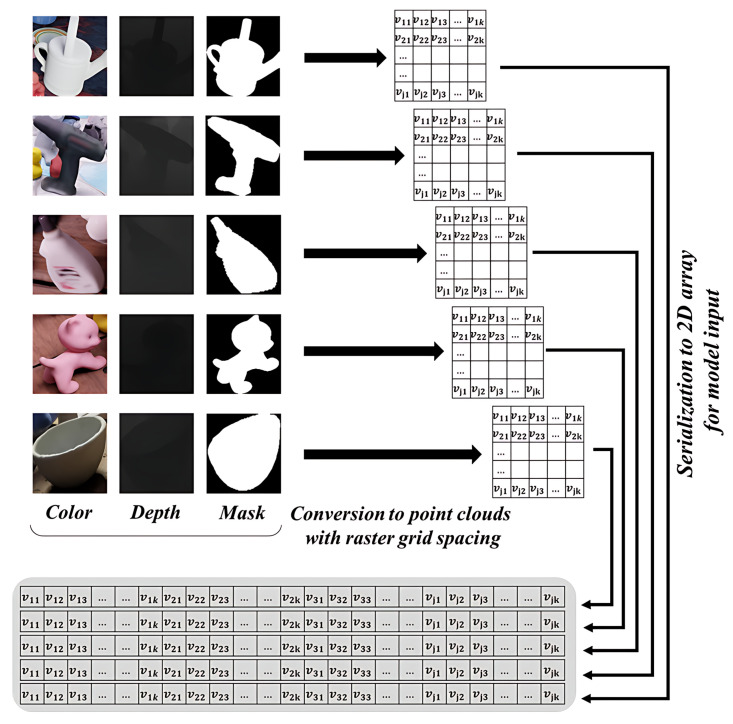
Method for extracting information about a plurality of objects from camera images using VFM and generating two-dimensional model input data.

**Figure 5:**
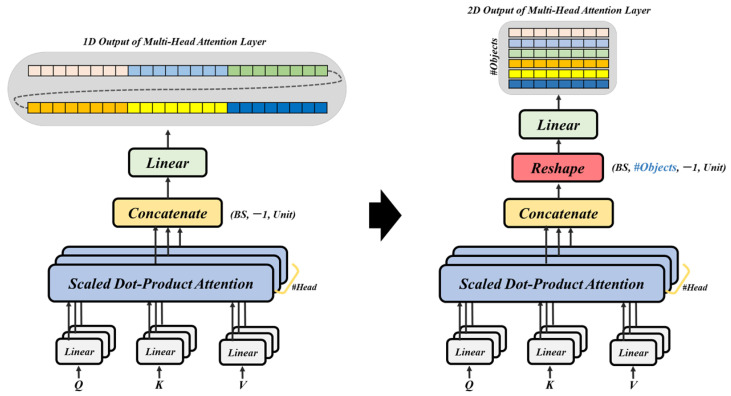
Modification of the output dimension of the extension of the attention layer.

This optimization requires adjusting the output dimension of the attention layer. We increase the output dimension to match the number of objects, thereby reducing the input size for the subsequent cumulative encoder layer. The computational load of the attention mechanism is effectively reduced by this dimension extension. This matrix dimension extension process for the output of the attention layer is illustrated in Figure 4 and Figure 5.

### 3.3. Low-Rank Weight Decomposition Adaptation Within the Attention Layer for Model Parameter Reduction

Transformer models improve inference performance by iteratively stacking encoder blocks, but this leads to an increase in model parameters. To enable efficient training while incorporating additional encoder blocks within constrained hardware resources, it is essential to reduce the previously expanded weight parameters.

Low-rank weight decomposition (LoRA) [28] is implemented in the linear blocks responsible for processing queries, keys, and values within the multi-head attention layer of the transformer model. This technique approximates the weight matrices in the linear blocks with two smaller matrices. By using low-rank matrices, LoRA effectively reduces the number of trainable parameters, resulting in lower computational cost, reduced memory consumption, and mitigation of overfitting during model training.

**Figure 6:**
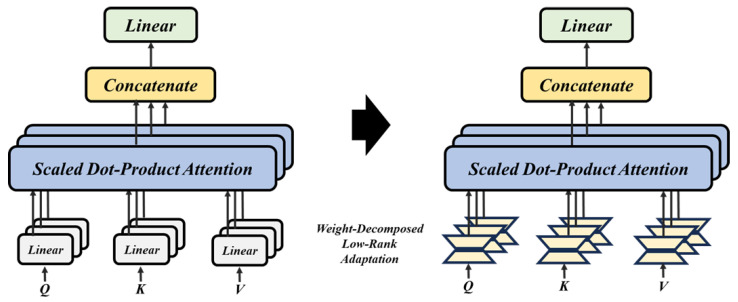
Modification of the adaptation of the low-rank weight decomposition within the attention layer.

In this study, the size of the number of columns in the front small matrix and the number of rows in the back matrix are divided by the number of heads in the multi-head attention. This scaling ensures that the dimensions of the matrices undergoing decomposition are appropriately adjusted for the specified number of attention heads. This process is illustrated in Figure 6.

### 3.4. Using Grouped Query Attention and RMS Normalization in the Transformer Model

Grouped query attention (GQA) [29] is an advanced mechanism designed to enhance the efficiency of transformer architectures by addressing the memory and computational constraints associated with traditional multi-head attention (MHA). By grouping queries and enabling shared key and value heads, GQA significantly reduces the total number of parameters and memory consumption while maintaining model accuracy.

RMS normalization [30] is a technique for adjusting the scale of input data in a neural network to improve the stability and performance of the learning process. RMS normalization is like the layer normalization used in previous transform models. However, RMS normalization differs in that it normalizes the input data by calculating the squared mean for each input dimension, adding a small constant, taking the square root, and then dividing. With fewer computations than layer normalization, RMS normalization speeds up the training of the model. It also stabilizes the learning process by scaling the input data and reducing sensitivity to the learning rate.

**Figure 7:**
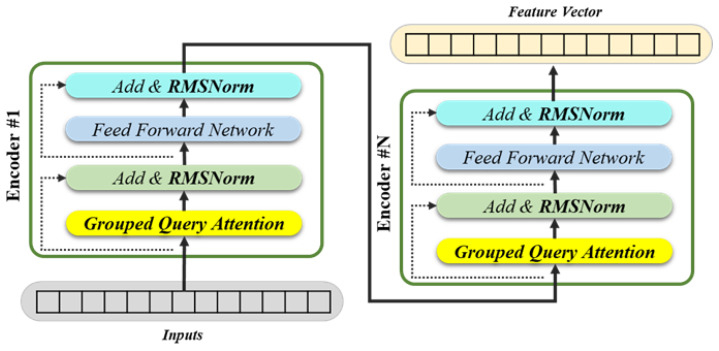
Modification of the application of the GQA and the RMS normalization to the transformer model.

In this study, we improved the attention layer by increasing the matrix dimension of its output layer to handle multiple objects, while also incorporating low-rank weight decomposition into the internal linear block. In addition, the GQA mechanism was applied, with the number of groups in GQA set as a multiple of 2 greater than the square root of the number of heads. The encoder normalization layer was changed from traditional layer normalization to RMS normalization, and the activation function in the feed-forward network was changed from ReLU to SiLU [31], as shown in Figure 7 below.

## 4. Experiments

### 4.1. Baseline Transformer Model for Single-Object Pose Estimation

The performance evaluations for the systematic improvement of the transformer model were conducted using the LINEMOD (LM) dataset [32]. This dataset is designed to support model-based training, detection, and pose estimation tasks in complex scenes, as well as the development and evaluation of techniques for detecting and estimating the six-degrees-of-freedom pose of texture-less 3D objects. The LM dataset consists of 15 video sequences, each with over 1100 frames of 15 different household objects with unique features such as color, shape, and size.

The hyperparameters of a baseline model of encoder-only transformer are shown in Table 2.

The hardware used for testing was Intel NUC 12 Extreme and NVIDIA RTX 4090 24G. In each test instance, the number of samples in the LM dataset used for training was 30,000. The number of steps per epoch was 100, the number of epochs was 300, and the batch size varied from 8 to 128.

The peak GPU memory usage of a model is affected by the batch size of the training data. The batch size, which represents the number of data samples processed in a forward and backward pass during training, plays a critical role in the model’s learning performance and efficiency. A smaller batch size can improve generalization by allowing more frequent updates to capture different patterns in the data, but it can also result in slower learning and increased loss variability. In contrast, a larger batch size stabilizes updates and takes advantage of parallel processing capabilities, although it may degrade generalization, increase the likelihood of convergence to a local minimum, and require more memory.

**Figure 8:**
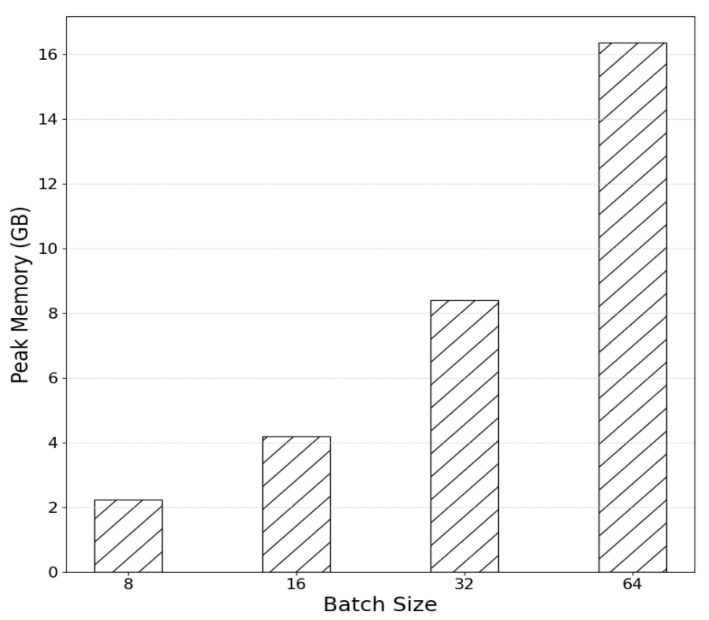
Comparison of peak memory usage relative to batch size in a baseline model.

The change in the peak memory usage as a function of batch size is shown in Figure 8 below. When the batch size is 64, approximately 16.34 G of GPU memory is used. If a larger batch size is applied, it will not run in this test hardware environment.

**Figure 9:**
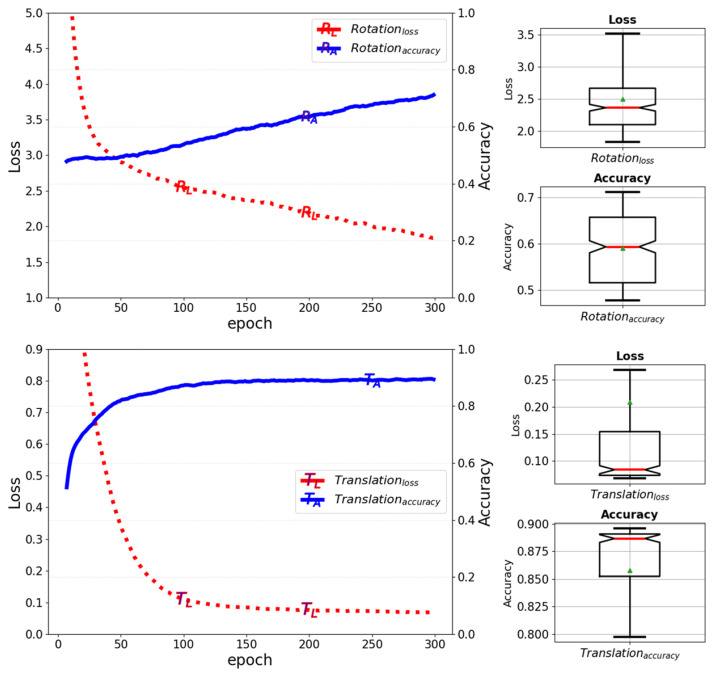
Training loss and accuracy for rotation and translation of the object pose in a baseline model.

Figure 9 below illustrates the variations in training loss and accuracy for the rotation and translation of the object pose of the baseline model when the batch size is set to 64 and the training epoch is run up to 300.

### 4.2. Extending the Matrix Dimension of the Attention Layer to Estimate Multi-Object Pose in a Single Prediction

We evaluated the peak GPU memory usage of the modified model that extends the matrix dimension of the attention layer output in the context of multi-object pose estimation. Increasing the matrix dimension axis raises the number of weight parameters, but reduces the complexity of the matrix dot product operation. This reduction in complexity leads to lower GPU memory usage during training, allowing for larger batch sizes to improve learning efficiency.

**Figure 10:**
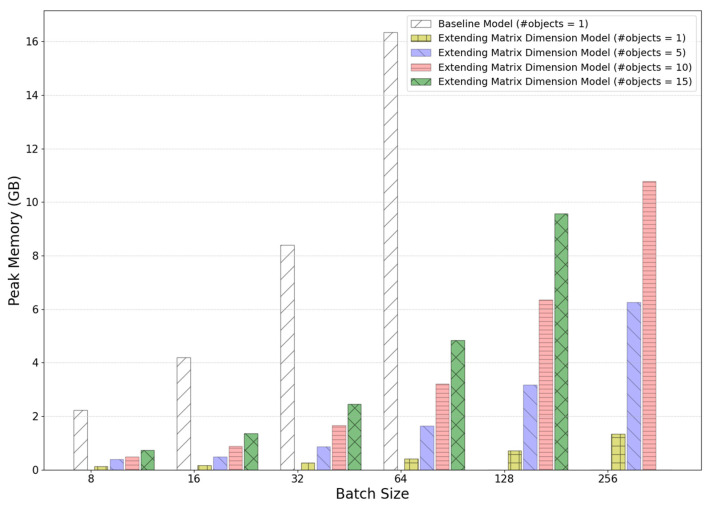
Comparison of the peak GPU memory usage as a function of the batch size and the number of objects for the pose estimation during a single inference.

Figure 10 shows the comparison of peak GPU memory usage based on batch size and the number of objects for pose estimation in a single inference. With a batch size of 64 and a single object, the peak memory usage of the baseline model was 16.34 GB, while the peak memory usage of the improved model was reduced to 0.41 GB, which is a significant reduction of 2.5% compared to the baseline model. When the number of objects increases to 15 with the same batch size of 64, the peak memory usage of the improved model is 4.84 GB, reflecting a reduction in GPU memory usage of approximately 75% compared to the baseline model.

For a single object, the execution time of each training epoch was 1.2 s per epoch, compared to 31 s for the baseline model. In the improved model, the execution time per epoch was 2.5 s while training 15 objects simultaneously. This is about 8% of the time required by the baseline model to train a single object.

The number of weight parameters in the improved model is 7,700,503, about 50% more than the number of parameters in the baseline model. The number of parameters is not affected by the change in batch size.

**Figure 11:**
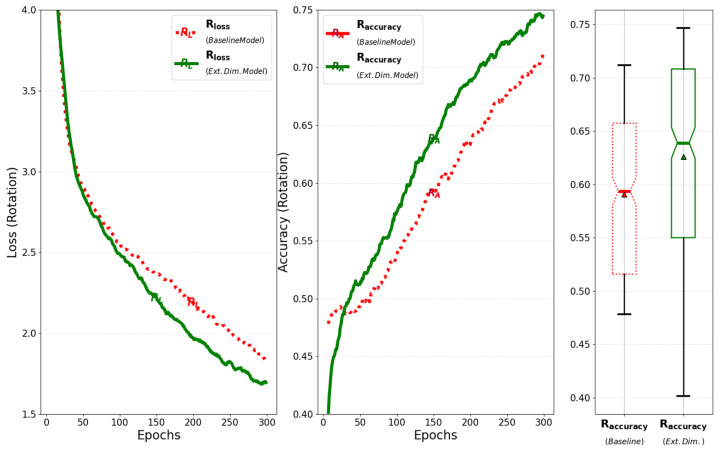
Comparison of training loss and accuracy for rotation between the baseline model and the model that extends the dimension of the matrix.

When the improved model is trained for 300 epochs with a batch size of 64, the training loss and accuracy of its rotation, which represent the model’s output, are compared to those of the baseline model, as illustrated in Figure 11. The results show that the improved model obtains a faster reduction in loss and a greater improvement in accuracy compared to the baseline model. This improvement not only accelerates loss reduction and improves accuracy but also optimizes GPU memory usage during training by extending the output axis of the attention layer in the encoder.

**Figure 12:**
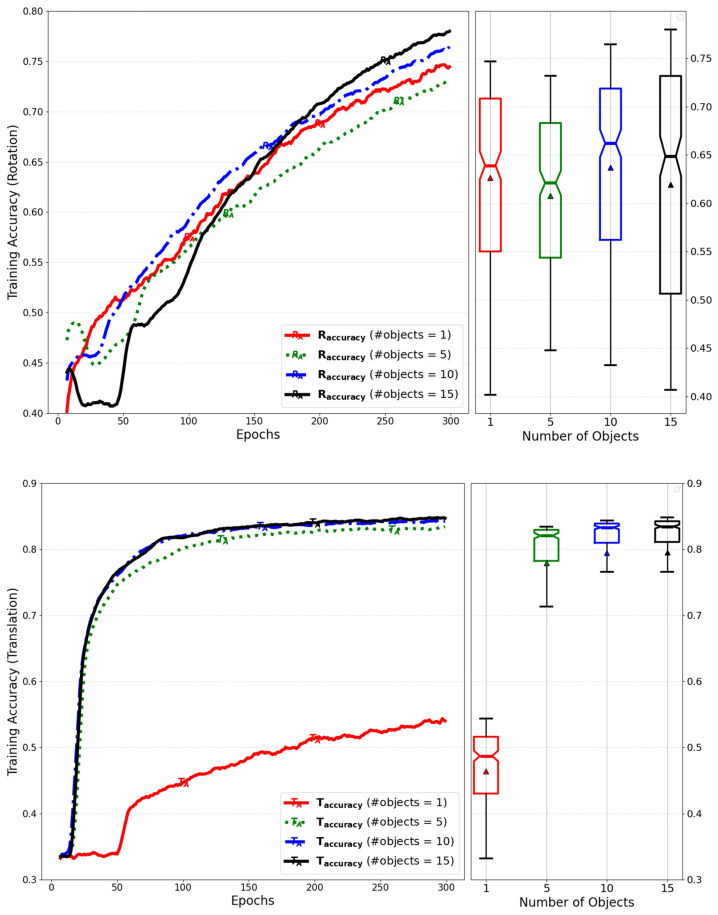
Evaluation of training accuracy for rotation and translation by variation of the number of objects during simultaneous inference by extending the dimension of the matrix.

Figure 12 shows a comparison of the training accuracy for rotation and translation, which was evaluated by varying the number of objects for the simultaneous inference.

### 4.3. Low-Rank Weight Decomposition Adaptation Within the Attention Layer for Model Parameter Reduction

**Figure 13:**
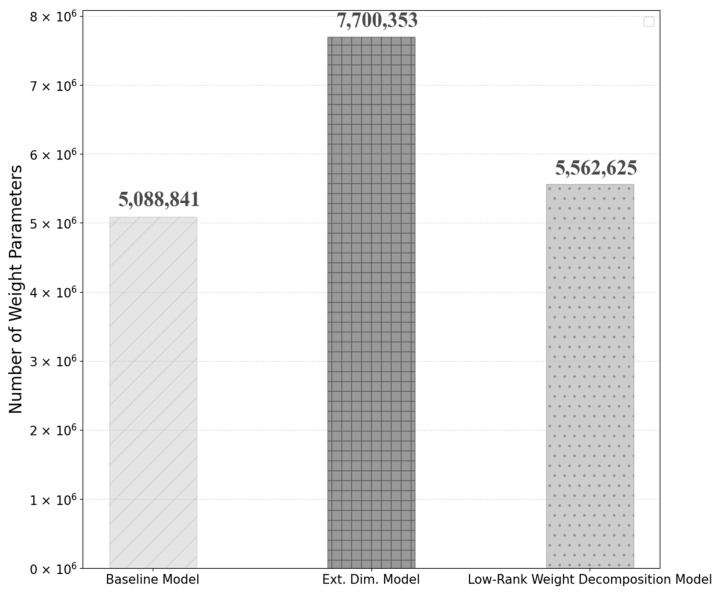
Comparison of the number of weight parameters between baseline, extending matrix dimension, and low-rank weight decomposition models.

To mitigate the increase in model weight parameters resulting from the dimensional extension of the output layer of the attention, a weight-decomposed low-rank adaptation is incorporated into the input linear block of the attention module. With this adaptation, the total weight parameters of the model are reduced to 5,562,625, which is about 28% less than the previous model, but about 9% more than the baseline model. This result is shown in Figure 13 below.

**Figure 14:**
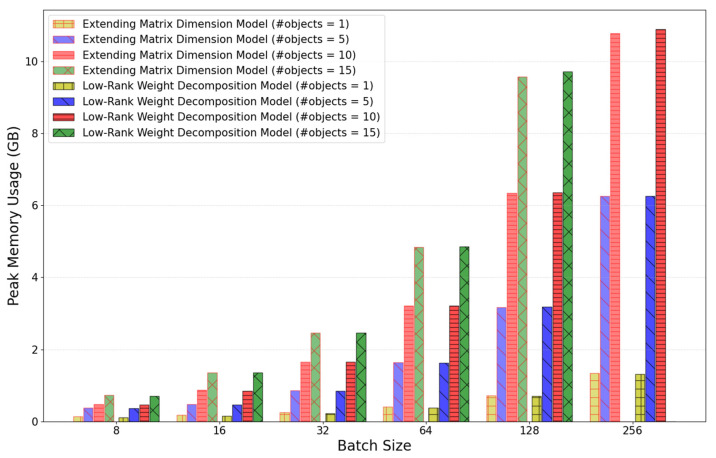
Comparison of the peak GPU memory usage in relation to the size of the batch and the number of objects between extending matrix dimension and low-rank weight decomposition models.

As shown in Figure 14 below, the number of weight parameters in the improved model has decreased, but the peak GPU memory usage has increased slightly to about 2% due to the addition of internal matrix multiplication.

**Figure 15:**
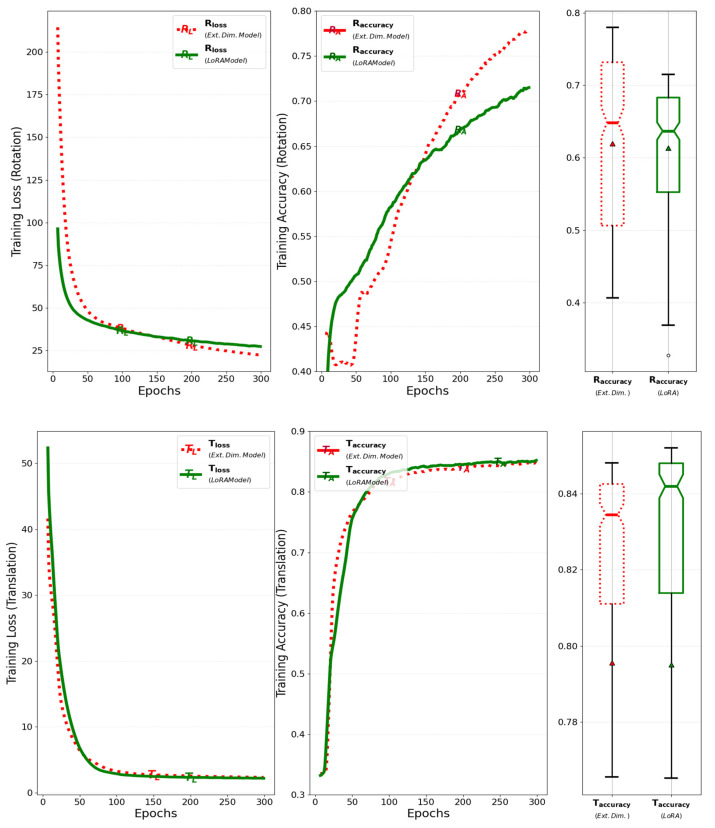
Comparison of training loss and accuracy for rotation and translation between the previous model and the model that adapts the low-rank weight decomposition within the attention layer.

The training loss and accuracy of the improved model, specifically for rotation and translation, were compared to those of the previous model when trained for 300 epochs with a batch size of 64. The incorporation of low-rank weight decomposition reduced the size of the matrices within the attention layer, leading to a reduction in the number of weight parameters, while increasing the depth of the model through the addition of matrices. As a result, the rotation accuracy decreased slightly during training, while translation accuracy improved, as shown in Figure 15. Despite the significant reduction in weight parameters, inference accuracy remained largely unchanged.

**Figure 16:**
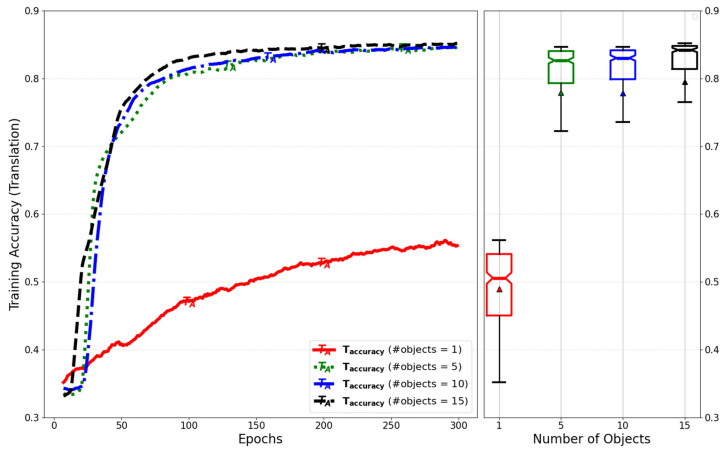
Evaluation of training accuracy for translation by number of objects for simultaneous inference of models with low-rank weight decompositions.

Figure 16 compares training accuracy for translation by adjusting the number of objects for simultaneous inference.

**Figure 17:**
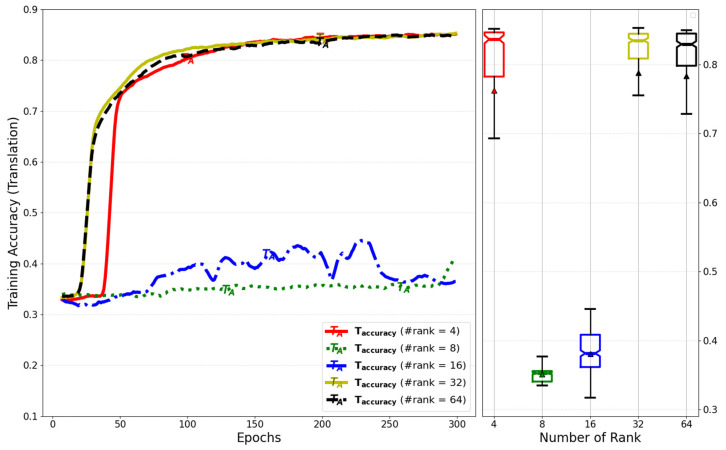
Evaluation of training accuracy for translation by number of ranks of weight decompositions.

Figure 17 shows a comparison of training accuracy for translation based on the number of ranks in the low-rank weight decomposition. To optimize the number of ranks while considering the model’s weight parameters, the ideal rank selection is equal to the number of linear units in the multi-head attention mechanism divided by the number of heads.

### 4.4. Using Grouped Query Attention and RMS Normalization in the Transformer Model

**Figure 18:**
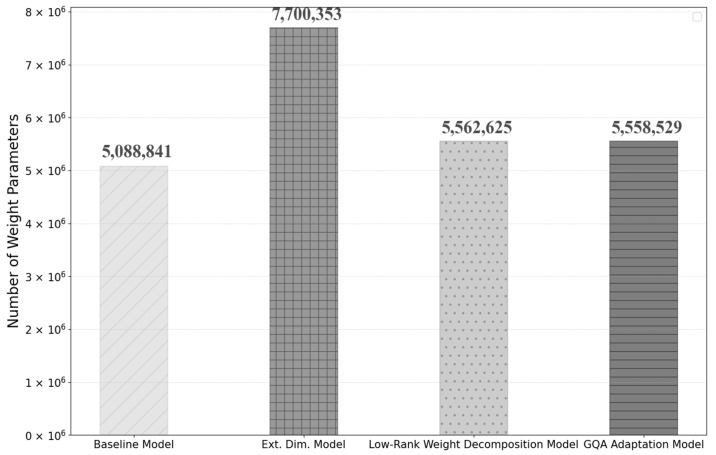
Comparison of the number of weight parameters between each model.

**Figure 19:**
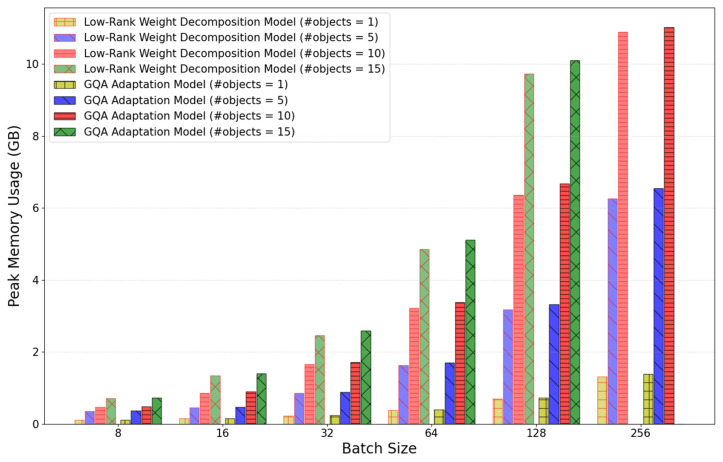
Comparison of the peak GPU memory usage in relation to the size of the batch and the number of objects.

The use of GQA and RMS normalization to improve model inference efficiency is also expected to improve training performance. Compared to previous models, no significant variations were observed in the number of weight parameters, GPU memory usage, or execution time per epoch during training, as shown in Figure 18 and Figure 19.

**Figure 20:**
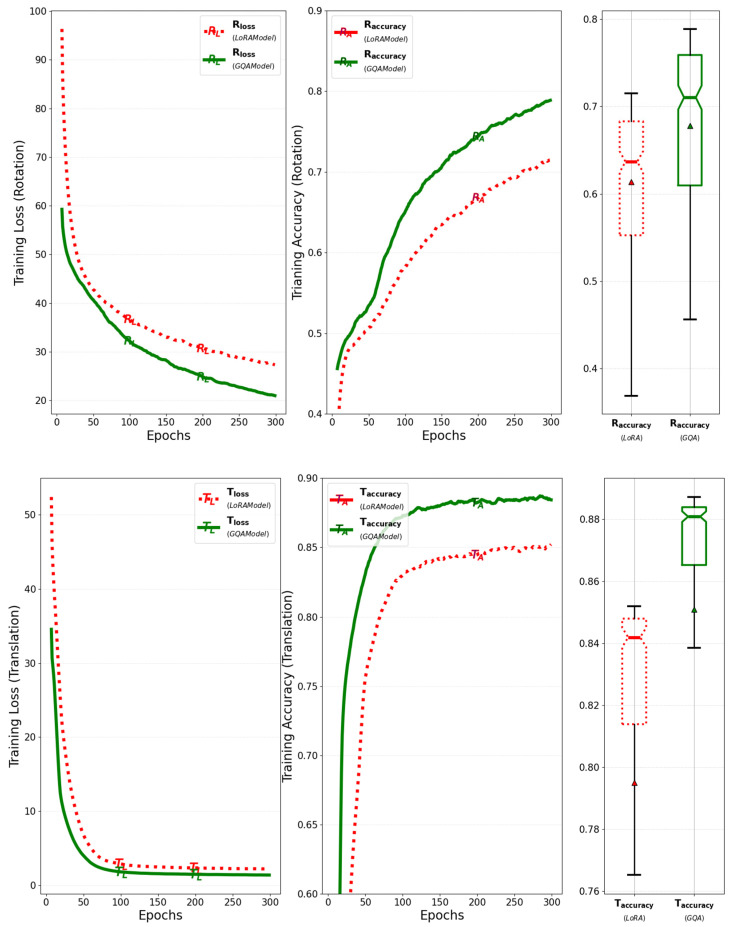
Comparison between the previous model and the model that adapts GQA and RMS normalization in terms of training loss and accuracy for rotation and translation.

The model with GQA and RMS normalization was trained for 300 epochs with a batch size of 64, and its training loss and accuracy for rotation and translation were evaluated against the previous model. The training accuracy for rotation improved by about 10%, and the training accuracy for translation improved by about 4% over the previous model when GQA and RMS normalization were applied. As shown in Figure 20, these results indicate a positive impact on loss convergence and overall accuracy improvement during training.

**Figure 21:**
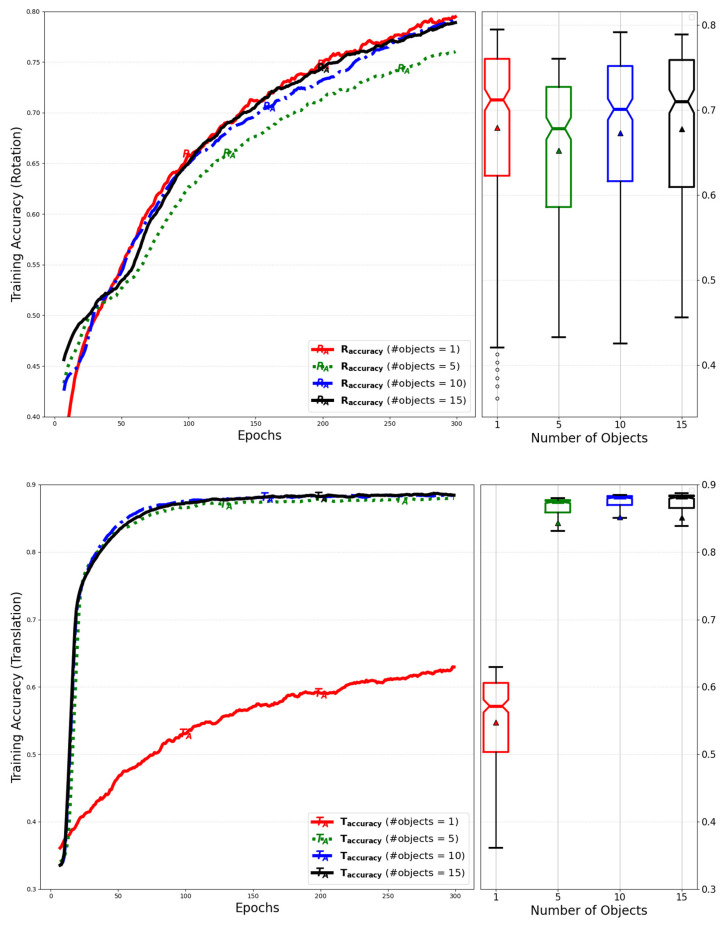
Evaluation of training accuracy for rotation and translation by number of objects for simultaneous inference of models with GQA and RMS normalization.

Figure 21 compares the training accuracy of rotation and translation while varying the number of objects for simultaneous inference. It can be observed that the training accuracy improves as the number of objects increases due to the higher amount of training per epoch.

**Figure 22:**
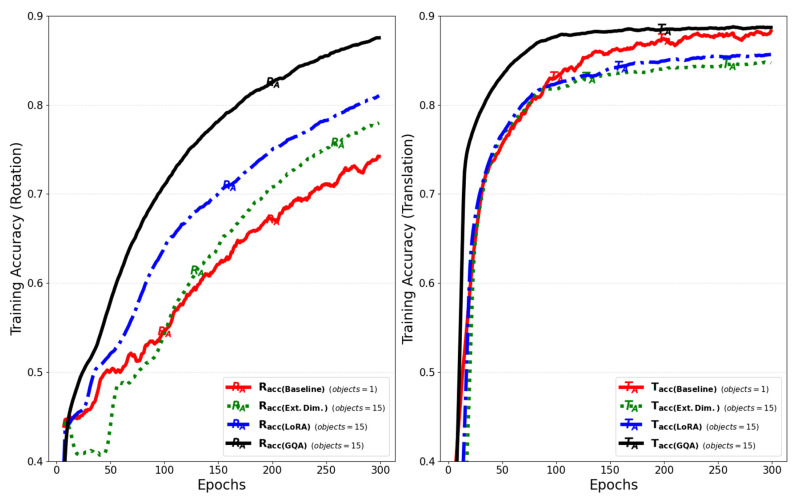
Changes in the training accuracy of the rotation and translation between the baseline model and the models with each improvement applied.

**Figure 23:**
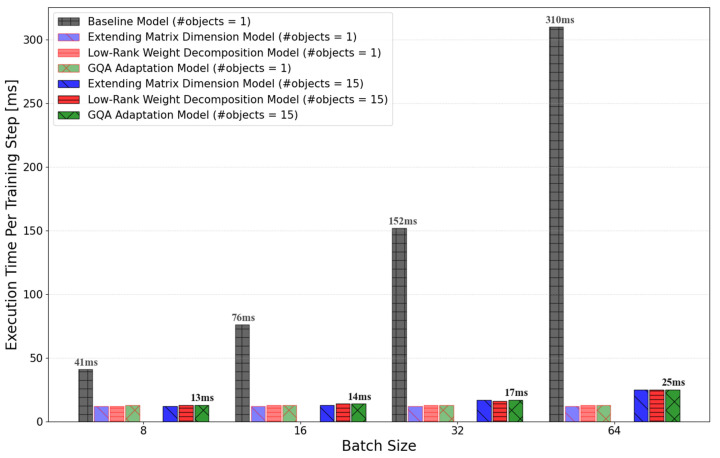
Changes in the execution time of the training step between the baseline model and the models with each improvement applied.

Starting from the baseline model, Figure 22 shows the change in the training accuracy for rotation and translation with the successive improvements. The reduction in computation time per training step with model improvements is shown in Figure 23.

## 5. Conclusions

Transformer architecture was originally designed for natural language processing and time series analysis but has since been utilized in generative models across multiple fields. It has proven to be an effective complement to RNN- and CNN-based approaches.

Object pose estimation refers to the process of identifying an object’s 3D position and orientation using image or sensor inputs. This method plays a vital role in robotic perception and interaction, facilitating operations like object manipulation.

We have incorporated a transformer architecture into a deep learning model for object pose estimation in computer vision. Using a vision foundation model, the input data is preprocessed by extracting object masks from color and depth images. Depth information is employed to generate a point cloud, which is treated as a sequential dataset.

The baseline model adopts a transformer architecture with an encoder-only implementation of the vanilla transformer. For multi-object pose estimation, input vectors corresponding to different objects are concatenated into a one-dimensional sequence. However, as the length of the input vectors increases, the model requires significant GPU memory for training. Consequently, the batch size cannot be increased significantly, limiting improvements in training efficiency.

We systematically improve the training efficiency while allowing for the simultaneous inference of multiple objects. First, we modify the output dimension of the transformer’s attention layer to optimize GPU memory usage during training. These improvements facilitate multi-object inference. Next, we incorporate low-rank weight decomposition into the linear input of the attention layer to reduce the model’s weight parameters while maintaining training accuracy. In addition, we refine the model for multi-object pose estimation by integrating GQA and RMS regularization, resulting in improved learning performance, faster loss convergence, and higher accuracy.

When evaluating the model with the implemented improvements, the peak GPU memory usage of the baseline model reached 16.34 GB while training on a single object. However, after incorporating the extended matrix dimension, the memory consumption dropped to 0.41 GB, which is approximately 2.5% of the memory consumed by the baseline model during training. The training execution time for the improved model was approximately 1.2 s per epoch, as opposed to 31 s for the baseline model.

By increasing the output dimension of the attention layer, the number of weight parameters in the model increased by approximately 50% relative to the baseline model. To address this issue, we implemented low-rank weight decomposition on the linear input layer of attention, which reduced the amount of parameter increase to about 9% relative to the baseline model.

Without changing the number of model weight parameters or GPU memory usage, the application of GQA and RMS normalization facilitated rapid loss convergence and improved training accuracy. As a result, training accuracy was improved by approximately 10% for rotation and 4% for translation.

These improvements in the transformer model have been evaluated in experiments using relatively low-performance hardware and small datasets. In the future, it will be necessary to compare performance using large datasets and batch sizes in environments with more GPU memory and powerful computing power.

## Figures and Tables

**Table 1:** Pseudocode for converting depth information into point clouds.

For each pixel (w, h) in the image: 1: Calculate the base point: pt_base = [w, h, 1] 2: Calculate the principal point offset: ppxy = [camera_ppx, camera_ppy, 0] 3: Calculate the inverse focal length matrix: fxy = [[1/camera_fx, 0, 0], [0, 1/camera_fy, 0], [0, 0, 1]] 4: Calculate the 3D point in camera coordinates: pt_vertex = (pt_base − ppxy) * fxy * depth_scale

**Table 2:** The hyperparameters of a baseline model of encoder-only transformer.

Multi-head attention	Linear units	256
	Number of heads	8
Feed forward network	Linear units	256
	Normalization	LayerNorm
Encoder-only transformer	Number of encoder block	4
	Number of weight parameter	5,088,841

## Data Availability

Data are contained within the article.

## References

[1] Vaswani A., Shazeer N., Parmar N., Uszkoreit J., Jones L., Gomez A.N., Kaiser L., Polosukhin I. (2017). Attention Is All You Need. arXiv.

[2] Lin K., Li L., Lin C.-C., Ahmed F., Gan Z., Liu Z., Lu Y., Wang L. SwinBERT: End-to-End Transformers with Sparse Attention for Video Captioning. Proceedings of the 2022 IEEE/CVF Conference on Computer Vision and Pattern Recognition (CVPR).

[3] Cao Q., Huang H., Liao M., Mao X. Ada-SwinBERT: Adaptive Token Selection for Efficient Video Captioning with Online Self-Distillation. Proceedings of the 2023 IEEE International Conference on Multimedia and Expo (ICME).

[4] Chen X., Zhao M., Shi F., Zhang M., He Y., Chen S. Enhancing Ocean Scene Video Captioning with Multimodal Pre-Training and Video-Swin-Transformer. Proceedings of the IECON 2023—49th Annual Conference of the IEEE Industrial Electronics Society.

[5] Dosovitskiy A., Beyer L., Kolesnikov A., Weissenborn D., Zhai X., Unterthiner T., Dehghani M., Minderer M., Heigold G., Gelly S. An Image Is Worth 16x16 Words: Transformers for Image Recognition at Scale. Proceedings of the International Conference on Learning Representations.

[6] Tan Z., Wang W., Shan C. (2024). Vision Transformers Are Active Learners for Image Copy Detection. Neurocomputing.

[7] Carion N., Massa F., Synnaeve G., Usunier N., Kirillov A., Zagoruyko S. (2020). End-to-End Object Detection with Transformers. Proceedings of the European Conference on Computer Vision—ECCV 2020.

[8] Huang Z., Tao X., Liu X. (2024). NAN-DETR: Noising Multi-Anchor Makes DETR Better for Object Detection. Front. Neurorobot..

[9] Liu X., Yang X., Shao L., Wang X., Gao Q., Shi H. (2024). GM-DETR: Research on a Defect Detection Method Based on Improved DETR. Sensors.

[10] Xu Y., Lin K.-Y., Zhang G., Wang X., Li H. RNNPose: Recurrent 6-DoF Object Pose Refinement with Robust Correspondence Field Estimation and Pose Optimization. Proceedings of the 2022 IEEE/CVF Conference on Computer Vision and Pattern Recognition (CVPR).

[11] Song C., Song J., Huang Q. HybridPose: 6D Object Pose Estimation under Hybrid Representations. Proceedings of the 2020 IEEE/CVF Conference on Computer Vision and Pattern Recognition (CVPR).

[12] Chen B., Chin T.-J., Klimavicius M. Occlusion-Robust Object Pose Estimation with Holistic Representation. Proceedings of the 2022 IEEE/CVF Winter Conference on Applications of Computer Vision (WACV).

[13] Zhang J., Wu M., Dong H. (2023). GenPOSE: Generative Category-Level Object Pose Estimation via Diffusion Models. arXiv.

[14] Khan Z., Khaquan M., Tafveez O., Samiwala B., Raza A.A. (2024). Beyond Uniform Query Distribution: Key-Driven Grouped Query Attention. arXiv.

[15] Chinnakonduru S.S., Mohapatra A. (2024). Weighted Grouped Query Attention in Transformers. arXiv.

[16] Cordonnier J.-B., Loukas A., Jaggi M. (2020). Multi-Head Attention: Collaborate Instead of Concatenate. arXiv.

[17] Jin P., Zhu B., Yuan L., Yan S. (2024). MOH: Multi-Head Attention as Mixture-of-Head Attention. arXiv.

[18] Lin X., Wang D., Zhou G., Liu C., Chen Q. (2023). TransPose: 6D Object Pose Estimation with Geometry-Aware Transformer. arXiv.

[19] Abdulsalam M., Aouf N. (2023). TransPose: A Transformer-Based 6D Object Pose Estimation Network with Depth Refinement. arXiv.

[20] Yu S., Zhai D.-H., Xia Y. (2024). CatFormer: Category-Level 6D Object Pose Estimation with Transformer. Proc. AAAI Conf. Artif. Intell..

[21] Li Z., Stamos I. Depth-Based 6DOF Object Pose Estimation Using SWIN Transformer. Proceedings of the 2023 IEEE/RSJ International Conference on Intelligent Robots and Systems (IROS).

[22] Cao C., Yu B., Xu W., Chen G., Ai Y. (2024). End-to-End Implicit Object Pose Estimation. Sensors.

[23] Caron M., Touvron H., Misra I., Jegou H., Mairal J., Bojanowski P., Joulin A. Emerging Properties in Self-Supervised Vision Transformers. Proceedings of the 2021 IEEE/CVF International Conference on Computer Vision (ICCV).

[24] Cheng T., Song L., Ge Y., Liu W., Wang X., Shan Y. YOLO-World: Real-Time Open-Vocabulary Object Detection. Proceedings of the 2024 IEEE/CVF Conference on Computer Vision and Pattern Recognition (CVPR).

[25] Kirillov A., Mintun E., Ravi N., Mao H., Rolland C., Gustafson L., Xiao T., Whitehead S., Berg A.C., Lo W.-Y. Segment Anything. Proceedings of the 2023 IEEE/CVF International Conference on Computer Vision (ICCV).

[26] Ravi N., Gabeur V., Hu Y.-T., Hu R., Ryali C., Ma T., Khedr H., Rädle R., Rolland C., Gustafson L. (2024). SAM 2: Segment Anything in Images and Videos. arXiv.

[27] Zou X., Yang J., Zhang H., Li F., Li L., Gao J., Lee Y.J. (2023). Segment Everything Everywhere All at Once. arXiv.

[28] Liu S.-Y., Wang C.-Y., Yin H., Molchanov P., Wang Y.-C.F., Cheng K.-T., Chen M.-H. (2024). DORA: Weight-Decomposed Low-Rank Adaptation. arXiv.

[29] Ainslie J., Lee-Thorp J., Michiel D.J., Zemlyanskiy Y., Lebrón F., Sanghai S. (2023). GQA: Training Generalized Multi-Query Transformer Models from Multi-Head Checkpoints. arXiv.

[30] Zhang B., Sennrich R. (2019). Root Mean Square Layer Normalization. arXiv.

[31] Elfwing S., Uchibe E., Doya K. (2017). Sigmoid-Weighted Linear Units for Neural Network Function Approximation in Reinforcement Learning. arXiv.

[32] Hinterstoisser S., Lepetit V., Ilic S., Holzer S., Bradski G., Konolige K., Navab N. (2013). Model Based Training, Detection and Pose Estimation of Texture-Less 3D Objects in Heavily Cluttered Scenes. Proceedings of the Asian Conference on Computer Vision—ACCV 2012.

